# 3D-Printed Anode for Power Generation and Wastewater Treatment in Microbial Fuel Cells

**DOI:** 10.3390/polym18060725

**Published:** 2026-03-17

**Authors:** Alfredo V. Reyes-Acosta, Natalia Orozco-Ordieres, Etelberto Cortez-Quevedo, Silvia Y. Martínez-Amador, Brenda V. Borrego-Limón, Francisco Alfonso Gordillo-Melgoza, José A. Rodríguez-de la Garza, Arturo I. Martínez-Enríquez, Pedro Pérez-Rodríguez

**Affiliations:** 1 Facultad de Sistemas, Universidad Autónoma de Coahuila, Ciudad Universitaria, Fundadores Km 13, Zona Centro, Arteaga 25350, Coahuila, Mexico; alfredoreyes@uadec.edu.mx; 2 Departamento de Ciencias del Suelo, Universidad Autónoma Agraria Antonio Narro, Calzada Antonio Narro 1923, Buenavista, Saltillo 25315, Coahuila, Mexico; natalia.orozcoordieres@gmail.com (N.O.-O.); etelberto.cortezq@uaaan.edu.mx (E.C.-Q.); 3 Departamento de Botánica, Universidad Autónoma Agraria Antonio Narro, Calzada Antonio Narro 1923, Buenavista, Saltillo 25315, Coahuila, Mexico; silvia.martinez@uaaan.edu.mx (S.Y.M.-A.); brendaborrego@uadec.edu.mx (B.V.B.-L.); 4 Departamento de Fitomejoramiento, Universidad Autónoma Agraria Antonio Narro, Calzada Antonio Narro 1923, Buenavista, Saltillo 25315, Coahuila, Mexico; francisco.gordillo@uaaan.edu.mx; 5 Departamento de Biotecnología, Facultad de Ciencias Químicas, Universidad Autónoma de Coahuila, José Cárdenas Valdez y Venustiano Carranza S/N, Colonia República Oriente, Saltillo 25280, Coahuila, Mexico; antonio.rodriguez@uadec.edu.mx; 6 Cinvestav Unidad Saltillo, Parque Industrial, Ramos Arizpe 25900, Coahuila, Mexico; arturo.martinez@cinvestav.edu.mx

**Keywords:** bioelectrochemical system, electrode, graphene, power density, coulombic efficiency

## Abstract

Microbial fuel cells (MFCs) are an emerging technology that converts the chemical energy stored in organic substrates into electrical energy using microorganisms as catalysts. However, their performance is often limited by the anode design and architecture. To address this, conductive anodes with well-defined pore sizes were manufactured via 3D printing and evaluated for electrical energy generation and wastewater treatment in microbial fuel cells. The maximum power density, coulombic efficiency, and accumulated biomass observed were 14.94 mW/m^2^, 4.87 ± 0.56%, and 0.186 ± 0.025 g, respectively, for the anode with a 2.3 mm pore size. The maximum chemical oxygen demand (COD) removal efficiency was 86.98 ± 1.89% for the anode with a pore size of 1.6 mm. However, this difference was minimal and not significant compared to the anode with a 2.3 mm pore size, which achieved 85.77 ± 2.31%. Additionally, the lowest internal resistance observed was 1246.44 Ω, corresponding to the MFC equipped with the anode with a pore size of 2.3 mm. Taken together, these results indicate that, when using 3D-printed anodes with controlled architectures, an intermediate pore size, neither too large nor too small, provides an adequate balance between electrochemical performance and efficient wastewater treatment in microbial fuel cells.

## 1. Introduction

Global urbanization has significantly increased municipal wastewater volumes. This growth creates major challenges for treatment and management. Conventional wastewater treatment is often energy-intensive and costly. Against this backdrop, microbial fuel cells (MFCs) have emerged as a promising technology that simultaneously treats wastewater and generates electricity [1,2]. MFCs rely on electroactive bacteria that oxidize organic matter, transferring electrons to the anode to produce electricity and degrade pollutants. However, MFC performance and scalability depend heavily on electrode properties, especially the anode, which supports microbial colonization and catalyzes substrate oxidation [3]. Conventional anodes, often limited by simple geometries and material restrictions, may reduce bacterial attachment. These limitations also decrease the available surface area for biofilm formation, ultimately decreasing electron transfer efficiency.

Three-dimensional printing (3DP), also known as additive manufacturing, enables the design of electrodes with complex architectures and controlled porosity. Through this technology, highly porous structures with large surface areas can be fabricated. Interconnected pathways form, which promote microbial colonization and improve mass transfer within bioelectrochemical systems [4,5,6,7].

Considering these advantages, several studies have explored the use of 3D-printed materials as electrodes in MFCs. For example, Bian et al. [8] manufactured a porous carbonaceous anode via 3D polymer printing (using a UV-curable resin), followed by a controlled carbonization process. The authors observed that the materials’ intrinsic biocompatibility and open porous structure led to a significant increase in microorganism metabolic activity, thereby enhancing the overall performance of the MFC. Similarly, Salvian et al. [9] fabricated an anode by stereolithography from carbonized resin (coated with furfuryl alcohol), exploring the potential of this material to enhance the performance of MFC-based biosensors for biochemical oxygen demand (BOD) monitoring. The results demonstrated that the fabricated anodes can be designed to enhance the dynamic range and sensitivity of MFC biosensors. On the other hand, Zhou et al. [10] developed 3D-printed anodes from stainless steel and titanium, both coated with polyaniline (PANI) via electropolymerization, and evaluated their performance in urine-fed microbial fuel cells (UMFCs). The authors found that the 3DP anodes considerably improved the electrochemical performance of UMFCs. Also, You et al. [11] investigated the use of a commercially available conductive polylactic acid (PLA) filament for the manufacture of an anode by 3DP. The authors evaluated this material in an MFC and observed stable power output despite the polymer’s low electrical conductivity. Finally, Constantino et al. [12] investigated stacking configurations of plant-microbial fuel cells (PMFCs) incorporating 3D-printed electrodes fabricated from a conductive PLA filament to evaluate their potential for bioelectricity amplification. Their results demonstrated that 3D printing can be effectively integrated into bioelectrochemical systems, enabling modular design, scalability, and field-oriented applications.

Despite recent advances in 3D-printed anode design, the specific effects of pore size and structural openness on energy generation and wastewater treatment in MFCs have not yet been fully characterized. Therefore, this study aims to determine how controlled pore size in 3D-printed anodes, fabricated with a conductive nylon-graphene filament, affects MFC performance. Electrical energy generation and organic matter removal were measured during wastewater treatment in the systems. Our findings provide a manufacturing method for low-cost, highly conductive, biocompatible, and scalable anodes to enhance the performance of BES.

## 2. Materials and Methods

### 2.1. Materials

The conductive 3D printing filament, composed of a blend of Nylon 12 and graphene oxide (GO), was procured from Color Plus 3D (Querétaro, Mexico). Sulfuric acid (95~98%), potassium dichromate (>99%), mercury sulfate (>98%), silver sulfate (>98%), and potassium biphthalate (>99.95%), utilized during the determination of chemical oxygen demand (COD), were purchased from JALMEK CIENTÍFICA (San Nicolás de los Garza, Mexico). The cation exchange membrane (CXM-200/CMI-7000S) was obtained from Membranes International Inc. (Ringwood, NJ, USA).

### 2.2. Fabrication of 3D Printed Anodes

**Figure 1:**
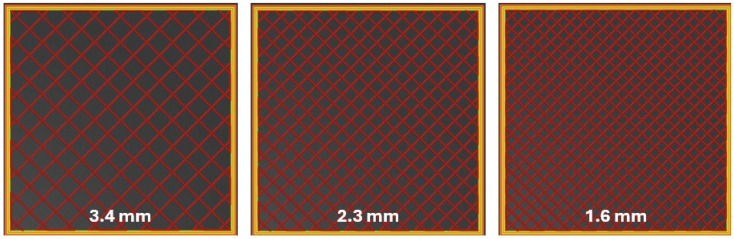
Different pore sizes in the Nylon 12/GO-synthesized anodes.

The anodes were manufactured on a Prusa i3 MK3S printer (Prague, Czech Republic) with a nozzle temperature set at 275 °C. The resulting Nylon 12/GO electrodes were thoroughly rinsed with distilled water and dried at room temperature for 24 h. All anodes had the same area (100 mm × 100 mm) and thickness (5.5 mm). Different pore sizes were evaluated: 3.4 mm, 2.3 mm, and 1.6 mm (Figure 1). These pore sizes were set by adjusting filament spacing in the digital design and verified with a digital caliper after fabrication.

### 2.3. Anode Characterization

The chemical structure and interfacial interactions of the conductive 3D printing filament were analyzed by Fourier-transform infrared (FTIR) spectroscopy using a Nicolet Magna 550 spectrophotometer (Nicolet Instrument Corporation, Madison, WI, USA) over a wavenumber range of 4000–400 cm^−1^. The morphology of the 3D-printed Nylon 12/GO composite was examined by scanning electron microscopy (JEOL JMS-7401F, JEOL Ltd., Akishima, Japan) at increasing magnifications. The electrical measurements of the composites were performed using the van der Pauw method [13] on square sheets with dimensions of 5 mm^2^ and a thickness of 0.1 ± 0.05 mm.

### 2.4. MFCs Construction and Operation

**Figure 2:**
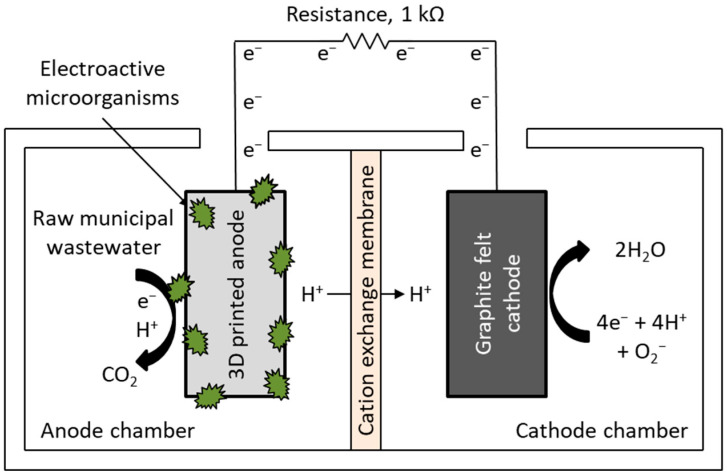
Schematic of the MFCs configuration.

Double-chamber MFCs (20 × 10 × 10 cm, 1000 mL working volume per chamber) were constructed as shown in Figure 2.

Each cathode chamber contained graphite felt (SinBarreraS, Saltillo, Mexico) (10 × 10 × 0.8 cm) as the cathode and 1000 mL of deionized water as the catholyte, aerated with a Lomas FL7950 pump (Ciudad de México, Mexico). Previously synthesized 3D-printed anodes were placed in the anode chambers, each of which was filled with 1000 mL of raw domestic wastewater serving as both the substrate and the microbial inoculum. No separate microbial cultivation occurred before MFC operation. Table 1 lists the physicochemical properties of the wastewater.

The anode and cathode chambers were separated by a cation exchange membrane, pre-hydrated in a 5% NaCl solution for 12 h at room temperature. The electrodes were positioned 4 cm apart, each 2 cm away from the membrane. An external resistance of 1 kΩ closed the cell circuit, with 316 L stainless steel wire (Master Wire Supply, Beavercreek, OH, USA) used as the electron collector. Cell voltage was monitored every 12 h for 27 days using a digital multimeter (Steren MUL-605, Ciudad de México, Mexico). A 15-day start-up period was implemented to allow acclimatization of the microbial community and promote the formation of an electroactive biofilm on the anode surface. Power density (*P_An_,* mW/m^2^) was calculated according to Equation (1):

(1) $$P_{An}=\frac{U^{2}}{R_{ext}\times A_{An}}$$

where *U* is the voltage (V), *R_ext_* is the external resistance (Ω), and *A_An_* is the projected anode area (m^2^) [14]. Additionally, the coulombic efficiency (*E_Cb_*, %) was calculated according to Equation (2):

(2) $$E_{Cb}=\frac{M\int_{0}^{t}Idt}{FbV_{An}\Delta COD}$$

where *M* = 32 is the molecular weight of oxygen, *I* is the electric current (calculated from the voltage generated by the MFC), *F* = 96,485.33 C/mol is Faraday’s constant, *b* = 4 is the number of electrons exchanged per mole of oxygen, *V_An_* is the volume of the substrate in the anode compartment (1 L), and *ΔCOD* is the difference in COD over time [14]. Chemical oxygen demand (COD) was determined at the beginning and end of the reaction to evaluate organic matter removal in the bioelectrochemical system using the spectrophotometric dichromate method according to the corresponding Mexican standard [15]. COD concentrations were obtained from the calibration curve of the method after spectrophotometric measurement. Finally, the biofilm biomass produced during wastewater treatment was quantified gravimetrically by measuring the increase in anode dry weight between the beginning and the end of the experiment.

### 2.5. MFCs Characterization

Polarization curves of the cells were determined using the variable resistance method, where the external resistance applied to the system was varied from 100 × 10^−3^ to 100 kΩ. The resulting power density was normalized to the total anode surface area.

## 3. Results

**Figure 3:**
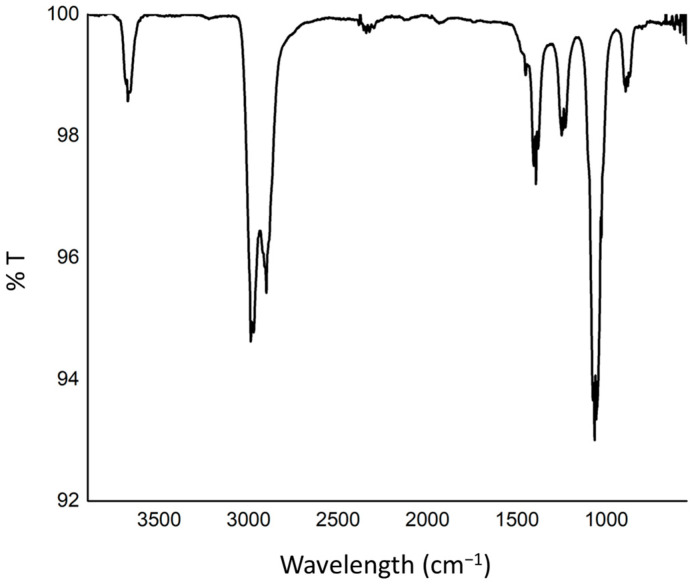
FTIR spectrum of the 3D-printed Nylon 12/GO filament showing characteristic Nylon 12 bands (N–H, amide I and II, –CH_2_– stretching) and C–O vibrations associated with GO, confirming composite formation and intermolecular interactions.

The FTIR spectrum (Figure 3) exhibits characteristic absorption bands associated with both Nylon 12 and GO, confirming the composite nature of the material. In the high-wavenumber region (3600–3000 cm^−1^), a weak but distinct absorption band centered at approximately 3300 cm^−1^ is observed, which is attributed to the N–H stretching vibration (amide A band) of Nylon 12. The presence of hydrogen bonding within the polyamide matrix typically broadens this band. A possible contribution from O–H stretching vibrations associated with hydroxyl groups in graphene oxide may also overlap in this region, suggesting intermolecular hydrogen bonding between the oxygen-containing groups of GO and the amide functionalities of Nylon 12 [16].

The strong absorption bands at approximately 2920 cm^−1^ and 2850 cm^−1^ correspond to asymmetric and symmetric stretching vibrations of aliphatic –CH_2_– groups, respectively. These peaks are characteristic of the long methylene sequences in the Nylon 12 backbone and confirm the preservation of the polymer’s aliphatic structure after compounding and filament fabrication. In the carbonyl region, a prominent absorption band observed at 1630–1650 cm^−1^ corresponds to the C=O stretching vibration of the amide I group of Nylon 12. Additionally, a band near 1540 cm^−1^ is assigned to the amide II vibration, arising from N–H bending coupled with C–N stretching. These bands are diagnostic of the polyamide structure and indicate that the secondary amide linkages remain chemically intact [17].

GO typically exhibits a C=O stretching band in the range of 1710–1730 cm^−1^ due to carboxyl and carbonyl groups. Broadening or partial overlap in this region may indicate interfacial interactions between the oxygen-containing functionalities of GO and the polymer matrix. The absence of new absorption bands suggests that no covalent bonding occurs during processing; rather, the interaction is predominantly physical, likely governed by hydrogen bonding and dipole–dipole interactions. In the 1460–1360 cm^−1^ region, absorption bands are assigned to –CH_2_– bending vibrations of Nylon 12. Strong absorptions in the 1200–1000 cm^−1^ range correspond to C–N stretching (amide III band) and C–O stretching vibrations. The relatively intense band near 1100–1050 cm^−1^ is particularly indicative of C–O stretching from epoxy and alkoxy groups in graphene oxide, further confirming the incorporation of GO into the polymer matrix [18].

In the fingerprint region below 1000 cm^−1^, bands associated with –CH_2_– rocking vibrations and skeletal vibrations of the polymer backbone are observed. Additional weak contributions in this region may arise from out-of-plane vibrations of graphene oxide sheets. Overall, the FTIR results confirm the successful incorporation of GO into the Nylon 12 matrix without significant chemical degradation of the polymer. The preservation of characteristic amide bands, together with the presence of oxygen-containing functional groups from GO, suggests that the composite structure is stabilized primarily through hydrogen bonding and secondary intermolecular interactions. These interfacial interactions may enhance filler dispersion and interfacial adhesion, contributing to improved mechanical integrity and electrical conductivity of the conductive 3D printing filament [19].

**Figure 4:**
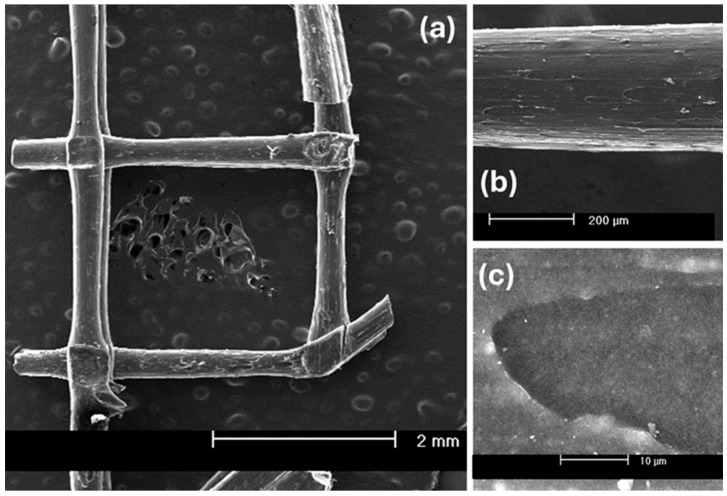
SEM images of the 3D-printed Nylon 12/GO composite at increasing magnification: (**a**) printed lattice structure (2 mm); (**b**) filament surface morphology (200 µm); (**c**) dense microstructure without significant GO agglomeration (10 µm).

SEM micrographs of the 3D-printed Nylon 12/GO composite are presented in Figure 4a–c. At low magnification (Figure 4a, scale bar: 2 mm), the printed lattice structure demonstrates good geometric fidelity and consistent filament deposition. The extruded strands exhibit uniform diameters and effective interlayer fusion at the filament intersections. No significant macroscopic delamination is observed, indicating adequate thermal bonding during the fused filament fabrication (FFF) process. Minor surface irregularities and localized voids are present, which may arise from printing parameters or the influence of GO on melt viscosity and flow behavior. At intermediate magnification (Figure 4b, scale bar: 200 µm), the filament surface appears relatively smooth, with subtle longitudinal striations aligned with the printing direction. These features are characteristic of shear-induced polymer chain orientation during extrusion. The absence of large agglomerates suggests relatively homogeneous dispersion of graphene oxide at the microscale. The compact morphology supports strong interlayer diffusion and mechanical integrity. At high magnification (Figure 4c, scale bar: 10 µm), the microstructure appears dense and continuous, with no evident microcracks or interfacial gaps. The lack of large GO aggregates indicates effective dispersion within the Nylon 12 matrix. Slightly textured or darker domains may correspond to embedded graphene oxide sheets. The absence of clear phase separation suggests strong matrix–filler compatibility, consistent with the hydrogen bonding interactions inferred from FTIR analysis [20,21].

The electrical conductivity of the printed composite material was measured at 20.7 ± 2.4 S/m using the van der Pauw method. This result confirms that the 3D-printed electrodes are conductive and suitable for microbial fuel cell applications.

**Figure 5:**
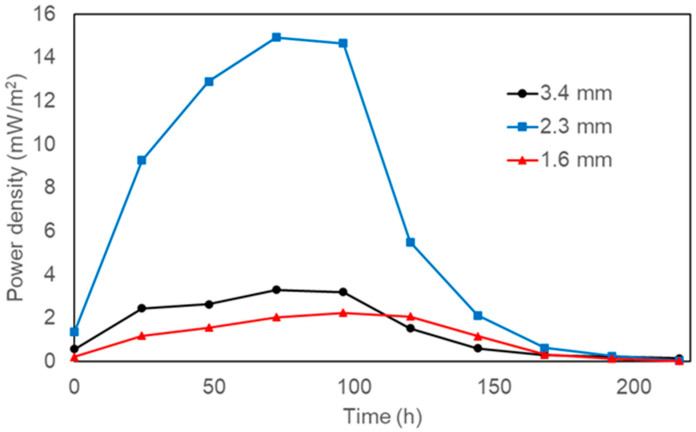
Power density as a function of time for MFCs equipped with 3D-printed anodes.

As shown in Figure 5, the maximum power density in the MFCs was 14.94 mW/m^2^ for the 3D-printed anode with a 2.3 mm pore size. This value was 6.7 times higher than with a 1.6 mm pore size (2.23 mW/m^2^) and 4.5 times higher than with a 3.4 mm pore size (3.30 mW/m^2^). This trend aligns with previous reports; for instance, Chong et al. [22] argued that very small pores can become biofouled by microbial growth, blocking the substrate (and nutrients) from reaching bacteria in the anode’s inner regions and thereby reducing the efficiency of converting organic matter to electricity. In contrast, Mei et al. [23] demonstrated that in large pores, the biofilm cannot evenly cover the space, leaving large gaps that slow and inefficiently transfer electrons. Collectively, these findings suggest that an intermediate pore size promotes more uniform biofilm development, enhancing electron transfer and, consequently, the electrical performance of the MFC.

**Figure 6:**
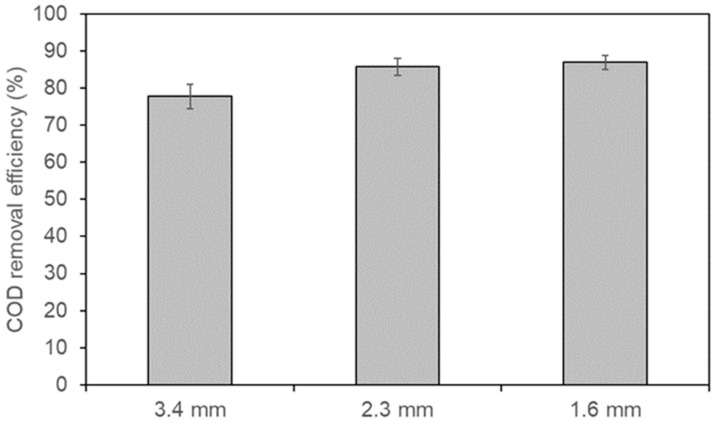
COD removal efficiency in MFCs equipped with 3D-printed anodes.

The anode pore size is a key parameter that influences organic matter degradation in bioelectrochemical systems, as it determines microbial loading capacity and substrate mass transfer [24,25]. Figure 6 presents the COD removal efficiencies obtained in the MFCs. The 3D-printed anodes with pore sizes of 3.4 mm, 2.3 mm, and 1.6 mm removed 77.76 ± 3.27%, 85.77 ± 2.31%, and 86.98 ± 1.89% of the organic matter available in the cells, respectively. These results suggest that decreasing the anode’s pore size increases the removal of organic matter in the system, possibly by increasing the effective surface area for biofilm development. However, the increase in removal efficiency is not directly proportional to pore size reduction; for example, the improvement from 2.3 mm to 1.6 mm is much smaller than that from 3.4 mm to 2.3 mm. This indicates a nonlinear trend and suggests a possible pore-scale saturation effect. Interestingly, although the smallest pore size showed the highest COD removal, the intermediate pore size (2.3 mm) produced higher coulombic efficiency and power density. Therefore, while smaller pores favor biofilm development and substrate degradation, an intermediate pore structure provides a better balance between surface area and mass transport, thereby enhancing electron transfer within the MFC.

**Figure 7:**
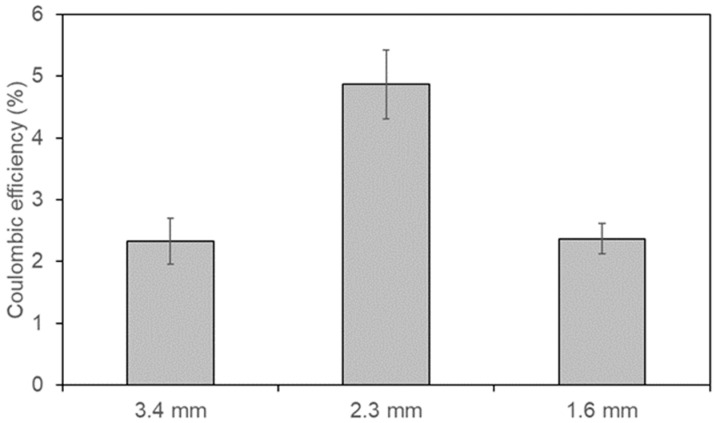
Coulombic efficiency of the MFCs investigated in this study.

Figure 7 shows the coulombic efficiency obtained in the MFCs equipped with the manufactured anodes. It can be observed that the anodes with pore sizes of 3.4 mm, 2.3 mm, and 1.6 mm achieved coulombic efficiencies of 2.32 ± 0.37%, 4.87 ± 0.56%, and 2.37 ± 0.25%, respectively. The highest coulombic efficiency was observed at an intermediate pore size of 2.3 mm, rather than at the smallest one. This result, consistent with the power density trend, indicates that greater COD removal efficiency does not necessarily lead to a higher electron recovery. This suggests that at a pore size of 2.3 mm, electroactive metabolism is favored, whereas at other pore sizes, non-electrogenic metabolic pathways likely dominate. Ren et al. [26] reported similar findings. They note that when pores are too small, protons generated by the microbiota during substrate oxidation can accumulate, lowering the pH of the support and significantly inhibiting the development of electroactive bacteria. Fan et al. [27] also argued that an appropriate anode pore size facilitates the capture and release of flavin molecules (endogenous mediators), thus accelerating electron transfer at the electrode.

**Figure 8:**
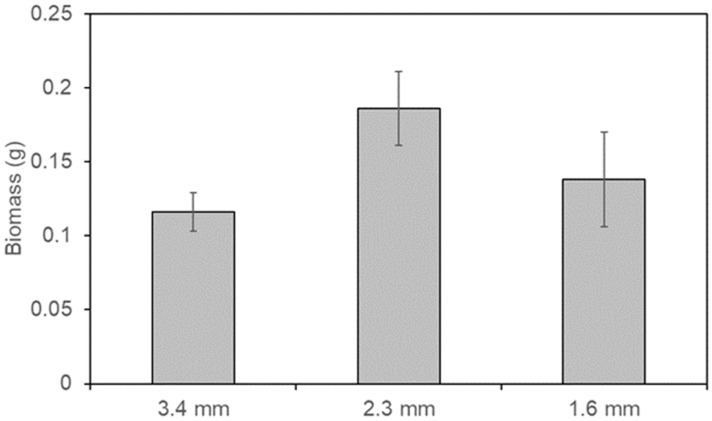
Biomass accumulation on 3D-printed anodes after wastewater treatment in MFCs.

Figure 8 shows the accumulated biomass on the 3D-printed anodes at the end of the experimental stage in the MFCs. Anodes with pore sizes of 3.4 mm, 2.3 mm, and 1.6 mm exhibited 0.116 ± 0.013 g, 0.186 ± 0.025 g, and 0.138 ± 0.032 g of biomass, respectively. These results indicate that an intermediate pore size (2.3 mm) yields the highest microbial retention and growth within the anode structure, potentially enhancing biofilm formation and improving MFC performance. This observation aligns with reports by other authors showing that anode architectures with moderate porosity, neither too large nor too small, positively influence bacterial colonization, mass transfer, nutrient transport, and biofilm stability [28,29], factors generally conducive to better MFC outcomes. However, as total biomass accumulation does not necessarily correspond exclusively to electroactive microorganisms or directly reflect electron-transfer efficiency, further analysis is required to determine the specific impact of biomass levels on MFC performance metrics.

**Figure 9:**
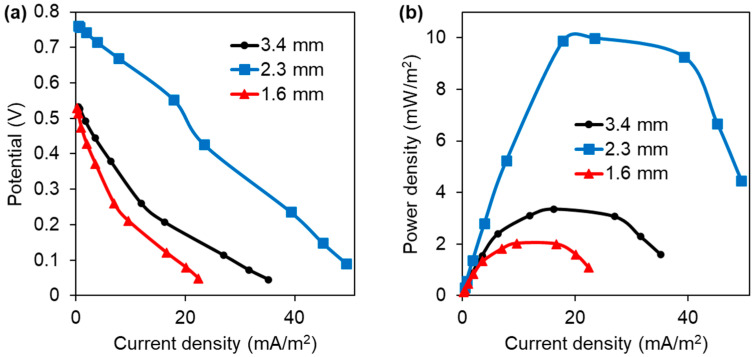
(**a**) Polarization and (**b**) power density curves as a function of current density in MFCs equipped with 3D-printed anodes.

Figure 9 shows the polarization and power density curves obtained from the MFCs. The internal resistance of each cell was estimated from the slope of the linear region of the polarization curve (Figure 9a) [30], yielding values of 1672.68 Ω, 1246.44 Ω, and 1613.59 Ω for MFCs equipped with 3D-printed anodes with pore sizes of 3.4 mm, 2.3 mm, and 1.6 mm, respectively. As shown in Figure 9b, the cell equipped with the intermediate-sized anode (2.3 mm) produced the highest power density and sustained higher power over a broader range of current densities than the other configurations. These results indicate that the anode with an intermediate pore size offers the most favorable electrochemical response among the tested architectures.

## 4. Conclusions

This study demonstrates that anodes with well-defined pore sizes, fabricated using 3D printing, can simultaneously enhance both electrical energy generation and municipal wastewater treatment in microbial fuel cells (MFCs). Among the tested architectures, the anode with an intermediate pore size (2.3 mm) achieved the highest performance, reaching a maximum power density of 14.94 mW/m^2^, a coulombic efficiency of 4.87 ± 0.56%, and a COD removal rate of 85.77 ± 2.31%. This configuration also showed favorable internal resistance and substantial biomass accumulation, indicating an optimal balance between electrochemical activity and microbial growth. These results underscore the practical potential of 3D printing for producing cost-effective electrodes and advancing scalable, energy-generating wastewater treatment for municipal applications.

## Figures and Tables

**Table 1:** Physicochemical characteristics of the raw municipal wastewater.

Parameter	Raw Municipal Wastewater
pH	8.32
Electrical conductivity	2293 µS/cm
Oxygen reduction potential	−221 mV
Total dissolved solids	1141 ppm
Chemical oxygen demand	963.33 mg/L
Temperature	26 °C

## Data Availability

The data that support the findings of this study are available from the corresponding author upon request.
